# Odorant-Binding Proteins OBP57d and OBP57e Affect Taste Perception and Host-Plant Preference in Drosophila sechellia

**DOI:** 10.1371/journal.pbio.0050118

**Published:** 2007-04-24

**Authors:** Takashi Matsuo, Shigeru Sugaya, Jyunichiro Yasukawa, Toshiro Aigaki, Yoshiaki Fuyama

**Affiliations:** Department of Biological Sciences, Tokyo Metropolitan University, Hachioji, Tokyo, Japan; Duke University, United States of America

## Abstract

Despite its morphological similarity to the other species in the Drosophila melanogaster species complex, D. sechellia has evolved distinct physiological and behavioral adaptations to its host plant *Morinda citrifolia,* commonly known as Tahitian Noni. The odor of the ripe fruit of M. citrifolia originates from hexanoic and octanoic acid. D. sechellia is attracted to these two fatty acids, whereas the other species in the complex are repelled. Here, using interspecies hybrids between D. melanogaster deficiency mutants and *D. sechellia,* we showed that the *Odorant-binding protein 57e (Obp57e)* gene is involved in the behavioral difference between the species. D. melanogaster knock-out flies for *Obp57e* and *Obp57d* showed altered behavioral responses to hexanoic acid and octanoic acid. Furthermore, the introduction of *Obp57d* and *Obp57e* from D. simulans and D. sechellia shifted the oviposition site preference of *D. melanogaster Obp57d/e^KO^* flies to that of the original species, confirming the contribution of these genes to D. sechellia's specialization to M. citrifolia. Our finding of the genes involved in host-plant determination may lead to further understanding of mechanisms underlying taste perception, evolution of plant–herbivore interactions, and speciation.

## Author Summary

Most herbivorous insects specialize on one or a few host plants; understanding the processes and genetics underlying this specialization has broad implications across biology. *Drosophila sechellia,* a fruit fly endemic to the Seychelles, feeds exclusively on the ripe fruit of *Morinda citrifolia,* a tropical plant commonly known as Tahitian Noni. Although other fruit flies never approach this fruit because of its toxins, D. sechellia is resistant and is actually attracted by the same toxins. D. sechellia is a close relative of *D. melanogaster,* an established model species of genetics. By comparing D. melanogaster and *D. sechellia,* we revealed that two genes encoding odorant-binding proteins, *Obp57d* and *Obp57e,* are not only involved in the fruit fly's taste perception, but can also change the behavioral response of the flies to the toxins contained in the fruit. By knowing how an insect's food preference is determined by its genes, we can gain insight into how insect lifestyles evolve and investigate whether such changes can lead to the formation of new species. We can also begin to understand how to manipulate insects' behavior by changing their preference for particular substances.

## Abstract 3

Hybrids of*Drosophila melanogaster* mutants and*D. sechellia* reveal genes involved in the behavioral difference that makes*sechellia* specialized to its host plant, with implications for understanding plant-herbivore interactions and speciation

## Introduction

Every animal must locate and identify sufficient food to meet its biological requirements. For herbivorous insects, this results in an endless battle with their host plants [1]. For example, some plants develop a chemical defense system that causes toxicity to generalist herbivores [2]. In response, generalist herbivores may then evolve a behavioral system to avoid such toxic plants. If an insect species acquires resistance to a plant toxin, however, it may reap an ecological advantage by gaining exclusive access to the toxic plant and may subsequently evolve as a specialist herbivore with a specific preference towards that plant. Such physiological and behavioral specialization plays an important role in the evolution of divergent ecological interactions between herbivores and their host plants. Nevertheless, it does not necessarily follow that ecological specialization for a particular host plant drives speciation of herbivores itself. Such specialization may not be sufficient to maintain divergence between populations at an early stage of speciation, in the face of potential gene flow via hybridization between evolving populations. The role of ecological specialization in speciation remains, therefore, to be proven [3]. Thus, it is necessary to identify the genes and molecular mechanisms responsible for ecological adaptation if we are to understand whether ecological adaptation can be a cause, or merely a consequence, of speciation [4].

Behavioral adaptation of herbivorous insects to their host plants involves the evolution of the chemosensory system [5–7]. With the recent identification of olfactory and gustatory receptors [8], knowledge of the genetic and molecular mechanisms of insect olfactory and gustatory system markedly progressed. Recent analysis of genomic information from several insect species has also revealed that the number of genes encoding these receptors varies considerably between species, indicating a close relationship between the genomic constitution of chemoreceptor gene families and the species-specific lifestyles of insects [9–11]. Thus, it is likely that the genes responsible for ecological adaptation are to be found among these receptor-encoding and receptor-related genes.

Genetic studies of *Drosophila* have also contributed to a substantial amount of our knowledge of “speciation genes” [4]. However, these studies have primarily focused on genes that cause reproductive isolation, and genetic analysis of ecological adaptation is relatively rare. This is, in part, due to the surprisingly limited information about *Drosophila* in the wild, compared with those flies reared in the laboratory as a sophisticated model system of genetics. In fact, we know little about their natural foods in the wild, except for a few species.

Drosophila sechellia is a specialist of *Morinda citrifolia,* which is commonly known as Tahitian Noni [12]. Although D. sechellia shows a preference for and resistance to the ripe fruit of *M. citrifolia,* its most closely related species, D. simulans and *D. mauritiana,* as well as *D. melanogaster,* are generalists and die upon contact with *M. citrifolia,* and thus avoid the fruit [13,14]. Because of genetic resources available for D. melanogaster and *D. simulans, D. sechellia* is an ideal organism with which to explore the genetics of ecological specialization. Analysis of quantitative trait loci (QTL) between D. sechellia and D. simulans has already identified the chromosomal regions responsible for the interspecies difference in resistance to the toxicity of M. citrifolia [15]. However, D. sechellia's preference for M. citrifolia was explained only by the transformation of olfactory sensilla resulting in an increase of the ab3 subtype that responds to the host odorant methyl hexanoate (MH) [16]. These findings successfully describe the present status of D. sechellia's specialization for *M. citrifolia,* but the evolutionary history, especially how an ancestral population started to use the toxic plant as its host, has been unexplained.

Here, for the first time, we have identified genes involved in D. sechellia evolution. These genes are responsible for the behavioral differences between species in their responses to hexanoic acid (HA) and octanoic acid (OA), the toxins contained in the ripe fruit of *M. citrifolia,* which give it its characteristic odor. Having identified the genetic factors constituting D. sechellia's adaptation to *M. citrifolia,* we are now able to discuss more confidently whether host-plant specialization can drive D. sechellia speciation.

## Results

### Mapping of Locus Responsible for Interspecies Difference in Avoidance of HA

We previously reported that the behavioral difference (preference/avoidance) between D. sechellia and D. simulans in response to HA, one of the main components of odor from the ripe fruit of *M. citrifolia,* is controlled by at least one gene on the second chromosome [17]. Further analysis of the introgression lines between D. sechellia and the D. simulans second chromosome marker strain *(net b sd pm)* indicated that the behavioral difference is linked to the marker *pm,* which is on the distal end of the right arm of the second chromosome (I. Higa and Y. Fuyama, unpublished data).

Considering the fact that the overall structure of the second chromosome is conserved between D. simulans and *D. melanogaster,* we mapped the locus in more detail using a series of D. melanogaster deficiency strains lacking a terminal part of the right arm of the second chromosome. Because D. sechellia's preference for HA is a recessive trait to D. melanogaster's avoidance [17], the interspecies hybrids between D. sechellia and D. melanogaster deficiency strains that lack a region containing the responsible gene(s) were expected to show the D. sechellia–like phenotype, i.e., preference for HA.

**Figure 1:**
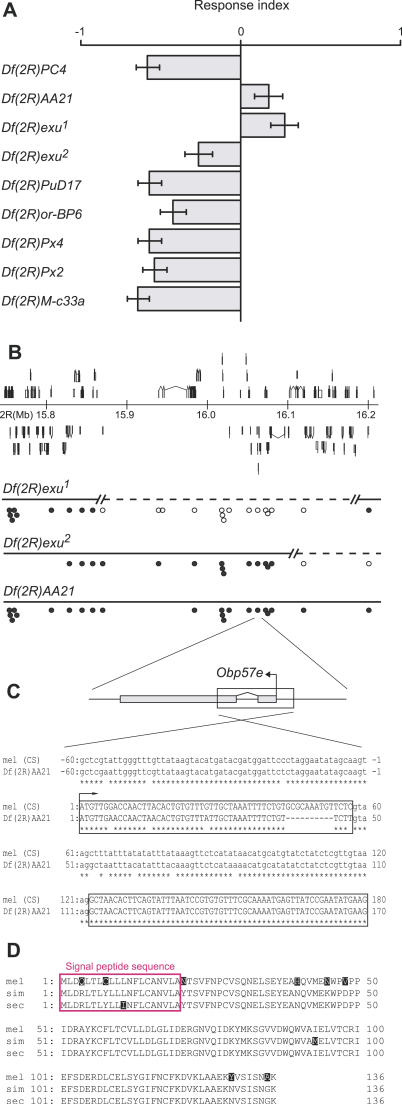
The Locus Responsible for Interspecies Difference in HA Avoidance Is Mapped to *Obp57e* (A) Behavioral screening of interspecies hybrids between D. melanogaster deficiency strains and D. sechellia. Response to HA was measured by the trap assay [17]. Response index (RI) = *(N_h_* − *N_w_)*/*(N_h_* + *N_w_),* where *N_h_* is the number of individuals trapped in 0.5% HA solution, and *N_w_* is that of individuals trapped in distilled water. Error bars indicate 95% confidence intervals determined by the binominal test of summed data from five replications of the assay with 100 females for each replication. (B) Determination of break points in deficiency chromosomes. A filled circle indicates that the deficiency-chromosome–derived sequence was detected, and an open circle indicates that the deficiency-chromosome–derived sequence was not detected at that position. (C) The *Df(2R)AA21* chromosome has a 10-bp deletion in the first exon of the *Obp57e* gene. A genomic sequence of *Df(2R)AA21* is aligned with that of the wild-type strain (CS). Predicted ORFs are boxed and capitalized. Arrows indicate the position and direction of translation start sites (ATG). (D) Comparison of *Obp57e* structure between D. melanogaster (mel), D. simulans (sim), and D. sechellia (sec). Predicted signal peptide sequence is boxed. Altered amino acid residues are highlighted.

Two deficiency strains, *Df(2R)exu^1^* and *Df(2R)AA21,* showed preference for HA when they were crossed with *D. sechellia,* defining the responsible locus within a very small chromosomal region, in combination with *Df(2R)exu^2^,* which showed avoidance to HA when crossed with D. sechellia (Figure 1A). Because the break points of these deficiency chromosomes had been deduced from cytological observations, we determined the position of these break points precisely by PCR-direct sequencing of genomic DNA from hybrids between D. melanogaster deficiency strains and D. sechellia (Figure 1B). According to the left break point of *Df(2R)exu^1^* and the left break point of *Df(2R)exu^2^,* the locus was narrowed down within about 200 kilobases (kb) of the genomic region that contains 24 predicted genes. There is no large deleted region in the *Df(2R)AA21* chromosome around this area, which is inconsistent with the result that *Df(2R)AA21* also showed preference for HA when crossed with D. sechellia. While examining the marker sequences used in break-point determination of *Df(2R)AA21,* however, we incidentally found that this chromosome has a small, ten–base pair (bp) deletion in the first exon (open reading frame [ORF]) of the *Odorant-binding protein 57e (Obp57e)* gene resulting in a frame-shift mutation (Figure 1C). Insect OBP is a protein secreted into the lymph of chemosensory hairs, and it has been shown to play a crucial role in chemosensation [18]. Thus, it seemed likely that *Obp57e* is a gene responsible for the interspecies difference in response to HA. However, when *Obp57e* ORF sequences from *D. melanogaster, D. simulans,* and D. sechellia are compared, there is no D. sechellia–specific alteration except for L11I, which does not affect the result of signal peptide–sequence prediction (Figure 1D). Thus, *D. sechellia Obp57e* ORF is supposed to be functionally intact, suggesting that the interspecies difference is not in the structure of the gene product, but rather in gene expression.

### Altered Expression Control of *Obp57e* in D. sechellia

**Figure 2:**
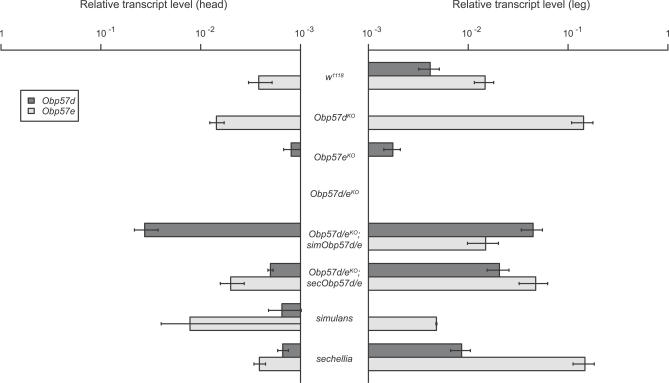
Quantitative RT-PCR Analysis of *Obp57d* and *Obp57e* Transcripts Heads and legs from 20 staged females were used for analysis. Transcript level relative to that of the ribosomal protein gene *rp49* is shown. Each bar represents the mean of three replicates. Error bars indicate standard error.

**Figure 3:**
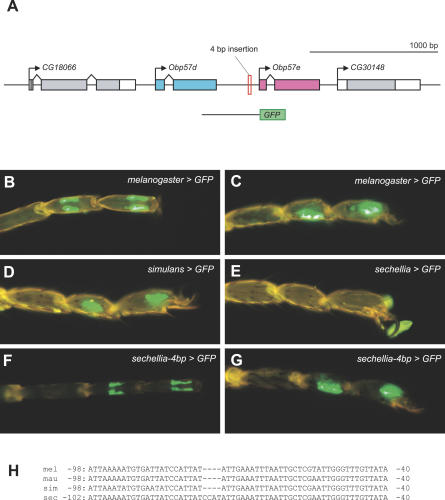
*GFP* Reporter Assay of *Obp57e* (A) Genomic structure around the *Obp57e* gene. Positions of the region used for the *GFP* reporter construct and 4-bp insertion in D. sechellia are indicated. Arrows on *Obp57d* and *Obp57e* indicate the position of the predicted translation-start sites (ATG), not that of the transcription start sites, which are unknown for these genes. (B–G) GFP reporter expression in tarsi. GFP driven by *D. melanogaster Obp57e* upstream sequence. (B) Dorsal view and (C) lateral view. GFP driven by (D) D. simulans and (E) *D. sechellia Obp57e* upstream sequence. (F and G) Removal of CCAT insertion from the *sechellia > GFP* construct restored GFP expression. (F) Dorsal view and (G) lateral view. (H) D. sechellia-specific 4-bp insertion in the upstream regions of *Obp57e*. Sequences from D. melanogaster (mel), D. simulans (sim), D. mauritiana (mau), and D. sechellia (sec) are aligned. Numbers indicate positions relative to the translation start site (ATG).

Quantitative reverse-transcriptase polymerase chain reaction (RT-PCR) analysis revealed that the level of *Obp57e* transcripts is higher in the legs of D. sechellia than in D. melanogaster and D. simulans (Figure 2). This could be due to an elevated transcription activity in particular cells and/or a widened expression pattern. According to the *lacZ* reporter experiment, *D. melanogaster Obp57e* is expressed only in four cells associated with chemosensory hairs on the fourth and fifth segments of each tarsus, the most terminal part of an insect leg [19]. We confirmed that as short as 450 bp of the upstream region of *Obp57e* completely reproduces the reported expression pattern (Figure 3A–3C). We then cloned the corresponding region from D. simulans and *D. sechellia,* and introduced it into D. melanogaster with a *green fluorescent protein (GFP)* reporter gene. The D. simulans sequence successfully reproduced the same expression pattern as observed in D. melanogaster (Figure 3D). However, the D. sechellia sequence failed to drive GFP expression in any parts of the fly body (Figure 3E), indicating that the function of the D. sechellia sequence to promote gene expression is altered. Indeed, when the upstream sequence of *Obp57e* is compared between species, a 4-bp insertion was found in the *D. sechellia Obp57e* upstream sequence (Figure 3H). GFP expression was restored by removing the inserted 4-bp nucleotides from the D. sechellia sequence, showing that this 4-bp insertion abolishes the function of the *D. sechellia Obp57e* promoter sequence in D. melanogaster (Figure 3F and 3G). Nevertheless, the results of *GFP* reporter experiments are inconsistent with that of quantitative RT-PCR analysis, thus, the exact expression pattern of *Obp57e* in D. sechellia remains unclarified. Therefore, it is necessary to evaluate using more direct methods whether *Obp57e* is truly responsible for the interspecies difference in behavioral response to HA.

### Targeted Mutagenesis of *Obp57d/e* Knock-Out Flies

**Figure 4:**
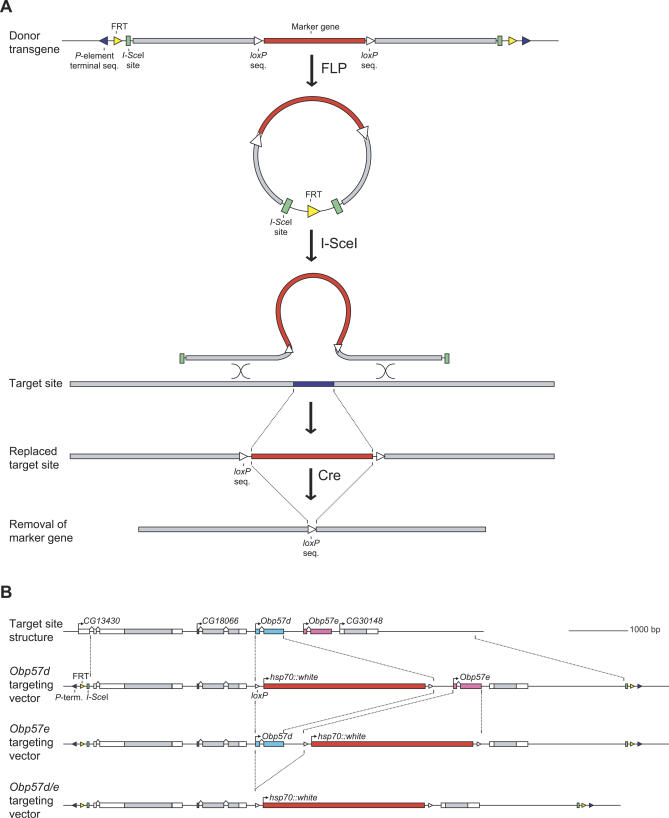
Generation of *Obp57d/e* Knock-Out Flies by Gene Targeting (A) Targeted gene replacement by the ends-out method. A donor transgene integrated into the other chromosome by P element–based transformation was excised by the FLP recombination enzyme at FLP recognition target (FRT) sites. Resulting circular DNA was linearized by I-SceI, inducing a precise replacement of a target gene with a marker gene. Finally, a marker gene was excised by Cre recombinase that recognizes *loxP* sequences, leaving a single 34-bp *loxP* sequence. (B) Vector structures for *Obp57d/e*-targeted mutagenesis.

We generated D. melanogaster knock-out flies for *Obp57e,* as well as for its neighbor *Obp57d,* and for both *Obp57d* and *Obp57e,* by gene targeting (Figure 4). The ends-out method was employed to achieve precise gene replacement in the gene-dense *Obp57d/e* region (Figure 4A). To avoid side effects on transcription of surrounding genes, the marker gene (3 kb) was excised by Cre recombinase, leaving only 34 bp of the *loxP* sequence. Each donor construct was designed such that the ORF was removed exactly from the ATG translation initiation site, but a putative poly-A additional signal was left intact, ensuring the termination of residual transcription that may affect the expression of downstream genes via read-through events (Figure 4B).

The loss of transcripts from the targeted gene was confirmed by quantitative RT-PCR in each knock-out strain (Figure 2). We observed, however, an unexpected interaction between *Obp57d* and *Obp57e* in their expression control. The amount of *Obp57e* transcripts was higher in *Obp57d^KO^* flies than in the *w^1118^* control strain. On the other hand, the amount of *Obp57d* transcripts decreased in the legs of *Obp57e^KO^* flies. Because each knock-out strain lacks the intron and the ORF, these regions may contain elements that influence the expression of the other gene.

### Altered Behavioral Responses to HA and OA in the Knock-Out Flies

**Figure 5:**
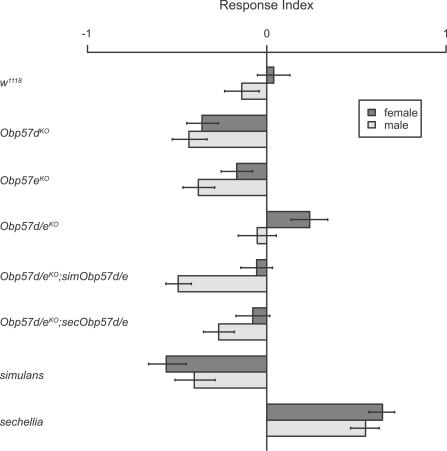
Olfactory Response of *Obp57d/e* Knock-Out Strains to HA in the Trap Assay Response index (RI) = *(N_h_* − *N_w_)*/*(N_h_* + *N_w_),* where *N_h_* is the number of individuals trapped in 1% HA solution, and *N_w_* is that of individuals trapped in distilled water. At least 300 individuals were tested in five replications of the assay. Error bars indicate 95% confidence intervals determined by the binominal test.

**Figure 6:**
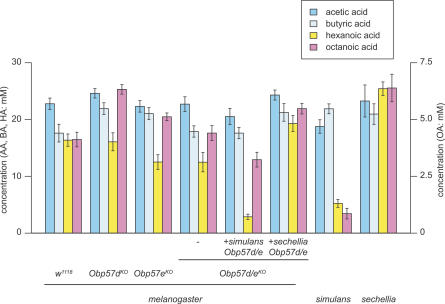
Preferred Concentration of Acids in Oviposition Site–Preference Assay Staged females were individually provided with four types of medium containing each acid at different concentrations. The concentrations were 0 mM, 10 mM, 20 mM, and 30 mM for acetic acid (AA), butyric acid (BA), and hexanoic acid (HA), and 0 mM, 2.5 mM, 5 mM, and 7.5 mM for octanoic acid (OA). The number of eggs laid on each medium was scored, and the weighted mean of acid concentration was calculated individually. Each bar represents a mean of 36 individuals from three replications. Error bars indicate standard error.

Each knock-out strain responded to HA differently from the control strain in the trap assay (Figure 5). *Obp57d^KO^* and *Obp57e^KO^* avoided HA, whereas females of *Obp57d/e^KO^* preferred it, suggesting that not only *Obp57e,* but also *Obp57d,* is involved in the behavioral difference observed in the screening assay. In fruit flies, host plants are largely determined by the oviposition site preference of adults. Thus, we also examined the oviposition site preference of knock-out flies in response to HA. Indeed, *Obp57e^KO^* and *Obp57d/e^KO^* seem to prefer lower concentrations of HA than the control flies, although the difference is not statistically significant (Figure 6, Tables 1–4). The direction of behavioral alteration was, however, not the same as that found in the trap assay for *Obp57d/e^KO^*. We also examined oviposition site preference in response to OA, the main toxic component in *Morinda* fruit. Because of its toxicity at high concentrations, the oviposition assay was carried out at concentrations lower than those of HA. *Obp57d^KO^* and *Obp57e^KO^* preferred higher concentrations of OA. This preference was particularly obvious for *Obp57d^KO^,* which was comparable to that of D. sechellia. Contrary to the responses to HA and OA, knock-out strains preferred concentrations of acetic acid and butyric acid similar to those preferred by control flies, showing that the alteration of behavioral responses in these knock-out strains is specific to HA and OA.

**Table 1:**
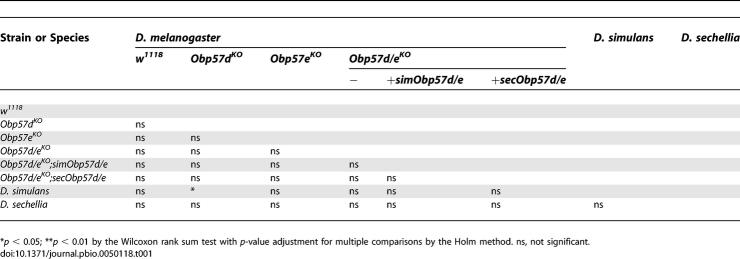
Statistical Analysis of Results of Oviposition Site–Preference Assay (Acetic Acid)

Our observation of the behavior of *Obp57d^KO^, Obp57e^KO^,* and *Obp57d/e^KO^* revealed that these strains are qualitatively different from each other in their responses to HA and OA. This strongly suggests that *Obp57d,* as well as *Obp57e,* is involved in D. sechellia's behavioral adaptation to M. citrifolia. Nevertheless, none of these knock-out strains was identical to D. sechellia in behavior. This is consistent with the results of quantitative RT-PCR analysis in which no knock-out strain exhibited an expression profile identical to that of *D. sechellia,* proving that this species is not a simple null mutant of *Obp57d* and/or *Obp57e*. Rather, D. sechellia seems to be a neomorphic mutant with an altered expression control of these genes.

### Replacement of *Obp57d/e* Region Altered Oviposition Behavior

To examine the functions of *Obp57d* and *Obp57e* in D. simulans and *D. sechellia,* we cloned these genes from D. simulans and D. sechellia and introduced them into the *D. melanogaster Obp57d/e^KO^* strain. Because an interaction between the two genes was observed with respect to their expression control, a genomic fragment spanning both *Obp57d* and *Obp57e* was used for genetic transformation. The resulting transformant flies showed altered responses to HA and OA in the oviposition site–preference assay (Figure 6; Tables 3 and 4). *Obp57d/e^KO^; simObp57d/e* flies avoided HA as D. simulans does. Conversely, *Obp57d/e^KO^; secObp57d/e* flies preferred high concentrations of OA as D. sechellia does. These results clearly showed that the *Obp57d/e* genomic region contains genetic information responsible for, at least in part, the interspecies differences in behavioral responses to HA and OA.

**Table 2:**
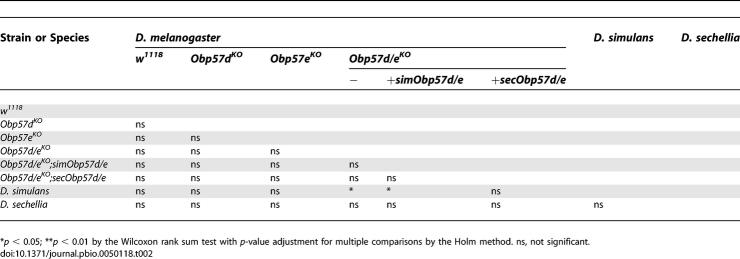
Statistical Analysis of Results of Oviposition Site–Preference Assay (Butyric Acid)

**Table 3:**
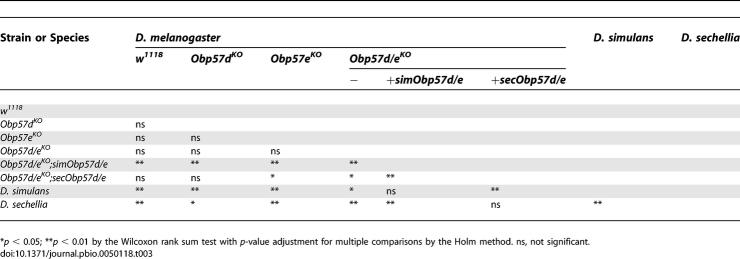
Statistical Analysis of Results of Oviposition Site–Preference Assay (Hexanoic Acid)

However, these transgenic flies are not complete mimicries of the original species. Although D. simulans avoided OA, as well as HA, the response of *Obp57d/e^KO^; simObp57d/e* flies to OA was not significantly different from that of the D. melanogaster control strain (Figure 6; Table 4). The responses of these two transgenic strains in the trap assay were also different from that of the original species (Figure 5). Consistent with the results of the oviposition assay, D. simulans avoided HA and D. sechellia preferred it. *Obp57d/e^KO^; simObp57d/e* females, however, did not avoid HA, and both sexes of *Obp57d/e^KO^; secObp57d/e* flies did not prefer it. Indeed, the expression profiles of *Obp57d* and *Obp57e* were not exactly the same between the transgenic strains and the corresponding original species (Figure 2). Although the genomic fragments seemed to reproduce the native expression better than the *GFP* reporters, there still remains significant differences in expression profile, particularly between *Obp57d/e^KO^; simObp57d/e* and D. simulans. These differences suggest a contribution of additional loci to *Obp57d/e* expression, and thus to the interspecies differences in behavioral responses to HA and OA.

Nevertheless, the *Obp57d/e* genomic region from D. simulans and D. sechellia could reproduce, at least in part, the behavioral pattern of the original species in an otherwise D. melanogaster genomic background, proving that a genetic difference in this region is actually involved in interspecies differences in behavioral responses to odorants contained in M. citrifolia. It should be particularly noted that the *Obp57d/e* region is alone sufficient for the strong avoidance of HA by *D. simulans,* which is a key trait in the evolution of D. sechellia's adaptation to *M. citrifolia,* as discussed below.

## Discussion

### Molecular Functions of OBP57d/e

LUSH (OBP76a), the best studied OBP in *D. melanogaster,* functions as an adaptor molecule in vaccenyl acetate (VA) recognition by an odorant receptor, OR67d [20]. Mutants lacking LUSH lose their neuronal response to VA; thus, they do not respond to VA behaviorally [18]. However, our *Obp57d/e^KO^* flies retained their behavioral responses to HA and OA, suggesting that OBP57d/e do not function as adaptors for HA and OA. Rather, they seem to modulate dose-dependent responses to HA and OA, which might be achieved by other proposed functions of OBP, such as the titration or degradation of ligands [21].

There are qualitative differences in the behavioral responses to HA and OA between *Obp57d^KO^* and *Obp57e^KO^* flies. These differences might reflect functional diversification between OBP57d and OBP57e. However, the elimination of either *Obp57d* or *Obp57e* affected the expression level of the other in these knock-out flies. *Obp57d* removal by gene targeting increased *Obp57e* expression level, and *Obp57e* removal repressed *Obp57d* expression. Thus, we cannot exclude the possibility that the behavioral differences between the knock-out strains are caused by an altered expression level of either gene. A more operative method such as the Gal4-UAS system could be used to separate promoters from ORFs, thus minimizing the interaction between these two genes in expression control. It would then be possible to examine the molecular functions of OBP57d and OBP57e independently.

### Expression Control of *Obp57d* and *Obp57e*

The results from our *GFP* reporter experiments and quantitative RT-PCR analysis are inconsistent. This inconsistency is also a feature of previous studies. Galindo and Smith [19] showed that the reporter constructs with 3 kb of upstream sequence from *Obp57d* and *Obp57e* were expressed in four cells in each leg, which matches the results of our *GFP* reporter experiments. However, using RT-PCR analysis, Takahashi and Takano-Shimizu [22] detected the transcripts not only in tarsi, but also in labella and wings. Together with the results of our quantitative RT-PCR analysis, it is clear that the reporter constructs do not reflect the complete expression pattern of *Obp57d/e*. The difference could be, at least in part, due to the lack of coding region in the reporter constructs. In fact, the elimination of a coding region of either *Obp57d* or *Obp57e* affected the expression level of the other gene in *Obp57d^KO^* and *Obp57e^KO^,* suggesting the involvement of ORFs and/or an intron in expression control (Figure 2). Furthermore, the introduction of the *Obp57d/e* genomic region from D. simulans and D. sechellia reproduced the expression of *Obp57d/e* in the head as well as in the legs, which was not observed in GFP reporter experiments.

Although the *Obp57d/e* genomic region contains a considerable part of the genetic information that controls *Obp57d/e* expression, it is still not sufficient to explain all the differences in the expression profile between the species; genetic factors at loci other than *Obp57d/e* are also likely to be responsible. There are two possibilities for such factors: (1) Trans-acting factors such as a transcription factor that is necessary for *Obp57d/e* expression, could control expression by determining which type of cell expresses *Obp57d/e,* or by determining transcription level in particular *Obp57d/e*-expressing cells. (2) Developmental factors determining the cell fate to become *Obp57d/e*-expressing cells, could increase/decrease the number of *Obp57d/e*-expressing cells by transforming cell fate at the expense of other cell types. In fact, ab1 and ab2 sensilla on antennae are transformed into ab3 sensilla in D. sechellia [16]. Such cell-type transformation might have occurred also in *Obp57d/e*-expressing cells. Genes of these two categories could be identified by, for example, screening of mutants that alter the *Obp57d/e* > *GFP* expression pattern.

### Genetic Factors Constituting D. sechellia's Adaptation to M. citrifolia

D. sechellia's adaptation to M. citrifolia consists of genetic changes at many loci. Although there are likely to be additional genetic differences between D. sechellia and *D. simulans,* the present status of D. sechellia's adaptation to M. citrifolia can be explained by alterations in three classes of genetic factors, as follows.

Factors responsible for resistance to the host-plant toxin OA: genes of this class are mapped to at least five loci scattered over all major chromosome arms [15], suggesting that the alleles at these loci were fixed independently from each other during the course of D. sechellia evolution.

Factors responsible for the olfactory preference for *M. citrifolia: D. sechellia* can detect *Morinda* fruit from as far as 150 m away [23]. Consistent with this observation, the number of olfactory sensilla specifically tuned to the host odor MH is increased in D. sechellia [16] (but also note that MH is commonly found in many other plants). In contrast, however, there are no data showing that D. simulans avoids *Morinda* fruit purely on the basis of olfactory cues; all behavioral assays, including our trap assay, enable flies to come in direct contact with the odor source. There is also no neural response to HA and OA from the antennae of either D. melanogaster or D. sechellia [16]. We therefore assume that the olfactory cues from *Morinda* fruit are fundamentally attractive to *Drosophila,* and not repulsive even to D. simulans. D. sechellia has an enhanced preference specifically tuned to the *Morinda* blend of olfactory cues, in which MH is a functionally major component. Genes responsible for this enhanced preference are thought to function in cell fate determination during neuronal development [16], but the number of genes involved is not yet known.

Factors responsible for the D. simulans' avoidance of *Morinda* fruit: we found this behavior to be based on gustatory cues, and confirmed that the introduction of the *Obp57d/e* region from D. simulans made D. melanogaster avoid HA in the same manner as D. simulans (Figure 6), proving that D. simulans' avoidance of HA-containing media as an oviposition site is determined by *Obp57d/e*. These two genes are physically close to each other and are thus treated as a single locus in the following discussions.

### Historical Order of Allele Fixation during the Course of D. sechellia's Evolution

Here, we discuss the order of allele fixation at the loci responsible for D. sechellia's adaptation to M. citrifolia. In particular, we focus on the issue of which mutation was the first to be fixed, because it must have played a key role in D. sechellia's evolution.

We speculate on this with respect to the ecological validity of each phenotype in light of natural selection. We assume that the first mutation arose at a single locus, and examine the resulting phenotype in an ecological context. (1) If the first mutation occurred at a resistance QTL, the resulting phenotype would be partially resistant to M. citrifolia. However, this phenotype is ecologically “silent” because these flies avoid *Morinda* fruit and may not lay eggs on it (a mutation on the resistance QTL cannot be advantageous unless a fly's behavior is changed). (2) If the first mutation was for the enhanced preference for the host odorant, flies should be attracted to *Morinda* fruit. This phenotype would conflict with the gustatory avoidance of *Morinda* fruit. The consequence of conflicting olfactory and gustatory cues is unpredictable, but we hypothesize that, at least in oviposition behavior, gustatory avoidance would override olfactory preference. Olfactory preference for a plant that is not acceptable as an oviposition site is ecologically inconsistent and obviously disadvantageous. (3) If the first mutation was at the *Obp57d/e* locus, the resulting phenotype would be the loss of gustatory avoidance of M. citrifolia. This seems to be also disadvantageous because flies would die upon contact with *Morinda* fruit because of their lack of resistance to it. However, there are circumstances that might enable an evolving population to survive and reproduce. The toxicity of *Morinda* fruit declines as it rots and OA degenerates [13]. Thus, there will be a point at which the toxicity is potentially low enough to be counteracted by the nutritional gain from the fruit. Moreover, because M. citrifolia flowers and fruits throughout the year, newly eclosing adults are likely to mate and reproduce on the same *Morinda* tree. Such conditions may not be optimal with regard to the quality of nutrients, but could potentially provide a niche with fewer competitors and may result in selection for a resistance to host toxicity. Altogether, among the three traits constituting D. sechellia's adaptation to *M. citrifolia,* only the loss of avoidance provides an ecologically realistic scenario for specialization without any other phenotypic changes.

The above discussion, of course, does not exclude the possibility of a simultaneous fixation of the alleles responsible for D. sechellia's adaptation to M. citrifolia. Nevertheless, it is parsimonious to assume that if there was a single causative mutation at an early stage of D. sechellia's adaptation to *M. citrifolia,* it was the mutation at the *Obp57d/e* locus that led to the loss of avoidance.

### Conclusion

*D. sechellia,* together with *D. mauritiana, D. simulans,* and *D. melanogaster,* serves not only as a subject of genetic analysis of reproductive isolation, but also as a good model for genetic analysis of ecological adaptation. There are more than 50 *Obp* genes in the D. melanogaster genome. Studies of natural variation at these loci will provide insight into the contribution of ecological interactions to the genomic constitution.

## Materials and Methods

### Fly preparation.

The fly strains used were *w^1118^* for *D. melanogaster,* S357 for *D. simulans,* and SS86 for D. sechellia [17]. Adult flies were collected immediately after eclosion, and staged for 3 d at 25 °C with a cotton plug soaked with liquid medium (5% yeast extract and 5% sucrose). Staged flies were used for the trap assay, the oviposition site–preference assay, and quantitative RT-PCR analysis.

### Trap assay.

A 30-ml glass flask containing 20 ml of HA solution in 0.05% Triton-X and a control flask containing the same amount of 0.05% Triton-X were placed in a plastic cage covered with a lid made of wire mesh. Up to 100 staged flies were introduced into the cage and kept in a dark, ventilated chamber overnight at 25 °C. The response index was calculated as RI = *(N_h_* − *N_w_)*/*(N_h_* + *N_w_),* where *N_h_* is the number of flies trapped in HA solution and *N_w_* is that of flies in the control trap.

### Determination of break points in deficiency chromosomes.

The PCR primers used are listed in Table 5. To amplify a fragment of about 300–600 bp from genomic DNA extracted from the interspecies hybrids between D. melanogaster deficiency strains and *D. sechellia,* each primer was designed within the ORF of predicted genes, with the expectation that there is enough conservation of sequences between the two species. PCR products were subjected to direct sequencing with the same primer used for amplification. The deficiency chromosome was considered to cover the position when the sequence derived from D. melanogaster or those from both D. melanogaster and D. sechellia were detected, and it was not considered to cover when only the D. sechellia sequence was detected.

**Table 4:**
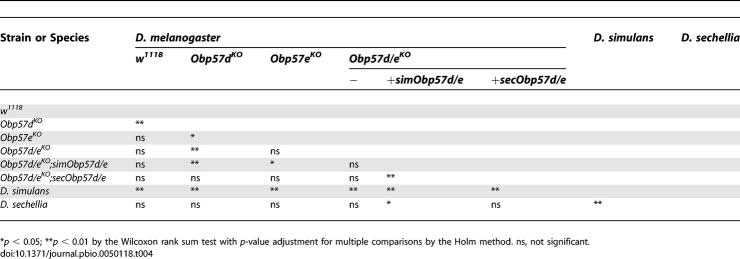
Statistical Analysis of Results of Oviposition Site–Preference Assay (Octanoic Acid)

**Table 5:**
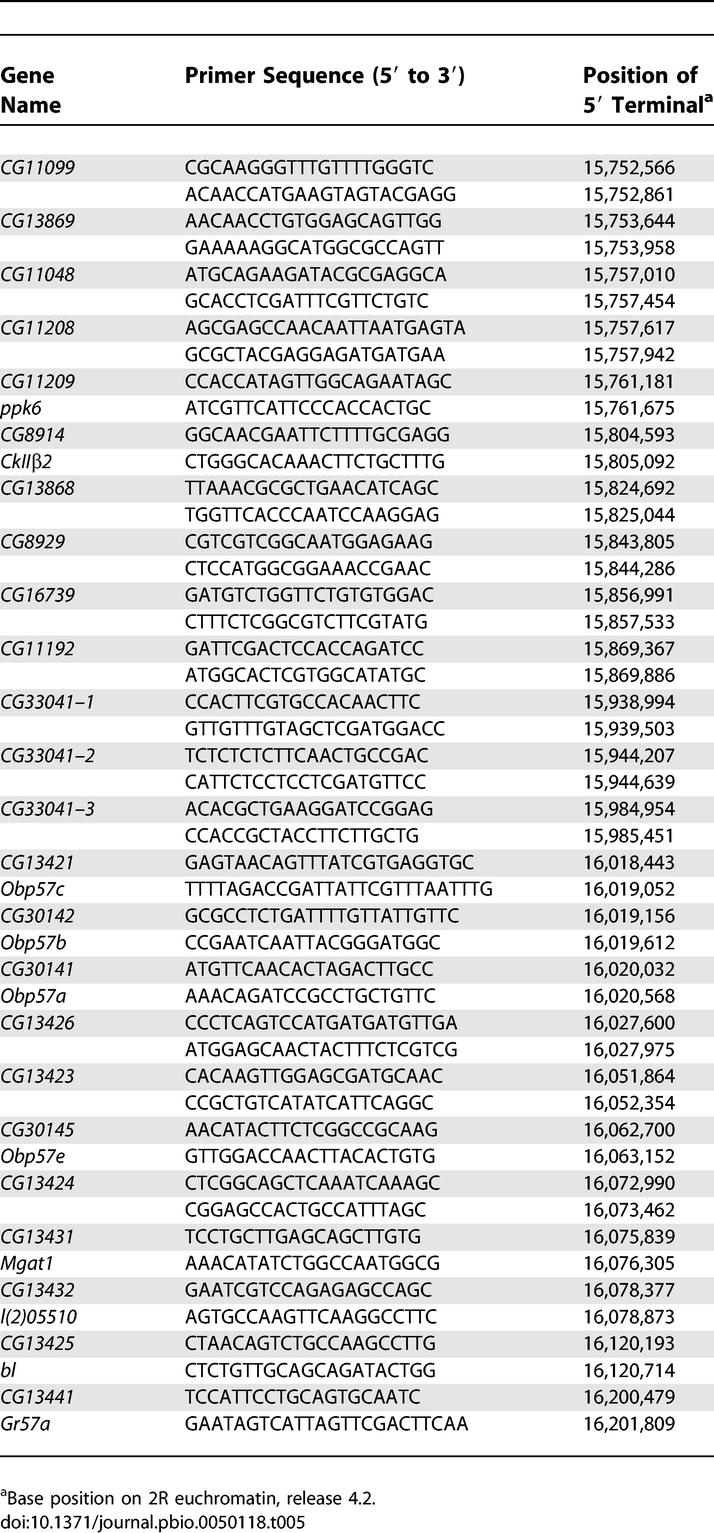
Primer Sequences Used for Determination of Break Points in Deficiency Chromosomes

### Signal peptide–sequence prediction.

Signal peptide sequence was predicted using SignalP 3.0 [24].

### GFP reporter analysis.

The genomic sequence upstream of *Obp57e* was PCR amplified with the primer pair 5′-(NotI) GCGGCCGC-GCGGTGGCACCCAAAATCAAT-3′ and 5′-(BamHI) AAAGGATCC-ACTTGCTATATTCCTAGGGAA-3′. PCR products were cloned into pGreenPelican [25], and then introduced into D. melanogaster by the established P element–based transformation method. To remove the inserted 4 bp from the *sechellia > GFP* construct, the vector was PCR amplified using the KOD-plus enzyme (Toyobo, http://www.toyobo.co.jp/e/) that does not append a T on the ends with the primers 5′-GATTATCCATTATATTGAAATTTAATTGC-3′ and 5′-ACATTTTTAATTGCACACACATTCAG-3′, and self-ligated after phosphorylation. At least five independent transformant lines for each construct were examined for GFP expression.

### Gene targeting.

Disruption of *Obp57d* and *Obp57e* was carried out by the ends-out method using the vectors provided by Dr. Sekelsky [26]. A *hsp70-white* marker gene was excised from pBS-70w with SphI and XhoI and subcloned into the SmaI site of pBSII after blunting to obtain pBSII-70w. The *Obp57d* upstream region amplified with the primer pair 5′-(EcoRI) AAAGAATTC-TTAATACGAGTATATCCCAGCAAAATCGAT-3′ (P1) and 5′-(BamHI-*loxP*) GGATCC-ATAACTTCGTATAGCATACATTATACGAAGTTAT-CAAACTAGTTGAAGATATCATAG −3′ and the downstream region amplified with the primer pair 5′-(PstI-*loxP*) CTGCAG-ATAACTTCGTATAATGTATGCTATACGAAGTTAT-GGACAAGTACTACGATACTGG −3′ and 5′-(NotI) GCGGCCGC-TATGAACACTCGCCGTGGTC-3′ (P2) were subcloned into pP{EndsOut2} with *hsp70-white* excised from the pBSII-70w with BamHI and PstI. The *Obp57e* upstream region amplified with the primer pair 5′-(BamHI-*loxP*) GGATCC-ATAACTTCGTATAGCATACATTATACGAAGTTAT-ACTTGCTATATTCCTAGGGAA −3′ and P1 and the downstream region amplified with the primer pair primers 5′-(PstI-*loxP*) CTGCAG-ATAACTTCGTATAATGTATGCTATACGAAGTTAT-GCGGCCGAGAAGTATGTTTC-3′ and P2 were subcloned into pP{EndsOut2}, similarly to the case of *Obp57d*. The *Obp57d* upstream region and the *Obp57e* downstream region were used for the *Obp57d/e* targeting vector. The fly transformation and targeting crosses were carried out as described by Sekelsky (http://rd.plos.org/pbio.0050118). Two, one, and three knock-out lines were obtained for *Obp57d, Obp57e,* and *Obp57d/e,* respectively. Each knock-out line was backcrossed to the *w^1118^* control strain for five generations.

### Introduction of *Obp57d/e* from D. simulans and *D. sechellia.*

Genomic fragments including *Obp57d/e* were PCR cloned from D. simulans and D. sechellia with the primers P1 and P2, and cloned into the pCaSpeR3 transformation vector. The *w^1118^; Obp57d/e^KO^* strain was transformed with these vectors by the established method. At least three independent transformant lines were obtained for each construct.

### Quantitative RT-PCR analysis.

RNA was extracted from the legs or heads of 20 staged females using an RNeasy Micro kit (Qiagen, http://www1.qiagen.com). cDNA was made using a SuperScript III First-strand Synthesis System (Invitrogen, http://www.invitrogen.com) with the oligo(dT)20 primer. Quantitative RT-PCR was carried out with the Chromo 4 realtime PCR analysis system (BioRad, http://www.bio-rad.com) using SYBR Premix ExTaq (Takara, http://www.takara-bio.com) with primers 5′-TTATTTTGGAAATTCAATTTAGAACTGCCG-3′ and 5′-TGATTCGGCTATATCTTCGTCTATTCCTTG-3′ for *D. melanogaster Obp57d,* 5′-TGCGCAAATGTTCTCGCTAACACTT-3′ and 5′-ATTCTCCATCACTTGGTGGGCTTCATA-3′ for *D. melanogaster Obp57e,* 5′- TTATTTTGGAAATTCAGTTTAGAATTTCCG-3′ and 5′- AATTGCTTCAGCTATATCTTCGTCTATTCC-3′ (P3) for *D. simulans Obp57d,* 5′- TGCGCAAACGTTCTTGCTTACACTT-3′ and 5′- GGCCATTTCTCCATCACTTGGTTG-3′ (P4) for *D. simulans Obp57e,* 5′- TTGGAAATTCAGTTTAGAAATTCTGAATGT-3′ and P3 for *D. sechellia Obp57d,* 5′- TGTGCGCAAATGTTCTTGCTTACACTT-3′ and P4 for *D. sechellia Obp57e,* and 5′- GCTAAGCTGTCGCACAAATG-3′ and 5′- TGTGCACCAGGAACTTCTTG-3′ for *rp49* of all species. Either of a primer pair was designed at an exon boundary to ensure amplification only from spliced transcripts.

### Oviposition site–preference assay.

Staged females were individually supplied with media (1% yeast extract [Gibco, http://www.invitrogen.com/content.cfm?pageid=11040]) and 0.8% Bacto Agar [Gibco]) containing an acid at four concentrations (0 mM, 10 mM, 20 mM and 30 mM for acetic acid, butyric acid, and HA; and 0 mM, 2.5 mM, 5 mM, and 7.5 mM for OA) simultaneously, and allowed the choice of medium for oviposition in a dark, ventilated box overnight at 25 °C. The number of eggs laid on each medium was scored, and the weighted mean of acid concentration was calculated for each individual. At least 36 individuals were tested for each strain with three replications.

## Supporting Information

### Accession Numbers

*Obp57d/e* sequence data have been deposited under the GenBank (http://www.ncbi.nlm.nih.gov/Genbank) accession numbers AB232138–AB232143.

## Abbreviations

bp: base pair
GFP: green fluorescent protein
HA: hexanoic acid
MH: methyl hexanoate
OA: octanoic acid
OBP: odorant-binding protein
ORF: open reading frame
RT-PCR: reverse-transcriptase polymerase chain reaction
